# Biases in Visual, Auditory, and Audiovisual Perception of Space

**DOI:** 10.1371/journal.pcbi.1004649

**Published:** 2015-12-08

**Authors:** Brian Odegaard, David R. Wozny, Ladan Shams

**Affiliations:** 1 Department of Psychology, University of California, Los Angeles, Los Angeles, California, United States of America; 2 Department of BioEngineering, University of California, Los Angeles, Los Angeles, California, United States of America; 3 Neuroscience Interdepartmental Program, University of California, Los Angeles, Los Angeles, California, United States of America; Imperial College London, UNITED KINGDOM

## Abstract

Localization of objects and events in the environment is critical for survival, as many perceptual and motor tasks rely on estimation of spatial location. Therefore, it seems reasonable to assume that spatial localizations should generally be accurate. Curiously, some previous studies have reported biases in visual and auditory localizations, but these studies have used small sample sizes and the results have been mixed. Therefore, it is not clear (1) if the reported biases in localization responses are real (or due to outliers, sampling bias, or other factors), and (2) whether these putative biases reflect a bias in sensory representations of space or *a priori* expectations (which may be due to the experimental setup, instructions, or distribution of stimuli). Here, to address these questions, a dataset of unprecedented size (obtained from 384 observers) was analyzed to examine presence, direction, and magnitude of sensory biases, and quantitative computational modeling was used to probe the underlying mechanism(s) driving these effects. Data revealed that, on average, observers were biased towards the center when localizing visual stimuli, and biased towards the periphery when localizing auditory stimuli. Moreover, quantitative analysis using a Bayesian Causal Inference framework suggests that while pre-existing spatial biases for central locations exert some influence, biases in the sensory representations of both visual and auditory space are necessary to fully explain the behavioral data. How are these opposing visual and auditory biases reconciled in conditions in which both auditory and visual stimuli are produced by a single event? Potentially, the bias in one modality could dominate, or the biases could interact/cancel out. The data revealed that when integration occurred in these conditions, the visual bias dominated, but the *magnitude* of this bias was *reduced* compared to unisensory conditions. Therefore, multisensory integration not only improves the *precision* of perceptual estimates, but also the *accuracy*.

## Author Summary

Almost all daily tasks performed by humans require localizing objects and events. Since spatial localization is critical for survival, it is expected that the brain performs this task accurately. We tested the accuracy of localizing simple sounds and sights in hundreds of human observers. We found that, on average, localizations of sounds and sights were *not* accurate, and observers made systematic errors: flashes of light were perceived to be *nearer* the center of the visual field than they actually were, and bursts of noise were perceived farther *away* from center. Surprisingly, computational analyses revealed that these biases in perceived location of sounds and sights are at least partly due to a bias in how sights and sounds are encoded by the sensory systems; the visual representation of the world is compressed towards the center and the auditory representation is expanded away from the center. When flashes and noise bursts were presented at the same location simultaneously and the observers perceived a common source for both stimuli, the bias in the perceived location was smaller, showing that synthesizing information across modalities helps increase accuracy in the perceived location of objects and events, compared to when only one sensation is available.

## Introduction

Nearly every function critical for human survival depends directly on accurate localization of objects and events in the environment. For example, our capacity to locate food, find potential mates, avoid predators, navigate new terrain, avoid obstacles, act upon objects, and orient towards sudden sounds requires a skilled aptitude to detect stimuli in the surrounding world. Spatial localization is thus a fundamental task that the brain has to solve at any given moment, and evolution has had millions of years to refine this function. Therefore, one would expect that the representation of space in all sensory modalities is generally accurate. However, the existing literature on human spatial localization contains many findings that are at odds with this assumption: representations of space in different sensory modalities appear to be biased, and no consensus yet exists regarding the direction and magnitude of the bias in a given modality.

For instance, while a majority of studies investigating localizations of simple visual stimuli that differ in azimuth have shown that visual localizations often show a bias towards the center of visual space [1–8], a few studies have reported peripheral biases or veridical perception [9–12]. In the auditory domain, findings of various studies have been mixed as well: some indicate that, on average, auditory localizations are often biased increasingly farther away from center as eccentricity increases [6,13–15], while others have reported central biases for average auditory localizations [16,17]. Thus, previous research has provided little consensus regarding the bias present in each sensory modality.

The previous investigations on spatial localization have used relatively small sample sizes, and consequently, variability in the reported findings could be due to sampling bias and/or the undue influence of outliers. Similar issues also apply to the previous reports of *precision* of spatial localization, particularly in the auditory domain. One study reported that the variability in auditory localizations remains relatively consistent across differences in azimuth [18], but two recent investigations found that auditory precision declines for peripheral targets [16,19].

Here, we present findings from an extraordinarily large dataset (384 subjects) to obtain more definitive answers regarding not only the direction and magnitude of biases in both the visual and auditory modalities, but also the precision in spatial localizations over a range of spatial positions. Previous speculations about the source of the variability in findings regarding the presence and direction of bias have focused on the specifics of the experimental paradigms, including the mechanism used to report location [11], the role of visibility of response location [20], the visibility of external boundaries [21], and the memory demands of the task as related to the time before response [8]. However, the variability in the results of previous studies could be a result of any of these factors or small sample sizes. In this study, we implement a simple yet rigorous paradigm to assess bias in which subjects’ localized briefly presented flashes of light and bursts of sounds; the response cursor was visible at all times to eliminate the influence of motor error, the locations of stimuli were unknown to subjects, the display was boundary-free, the response delay was minimal, and hundreds of subjects participated, all in an attempt to capture the true perceptual biases present in each modality. By analyzing responses from our immense dataset, we aim to clarify discrepant results in the literature regarding the accuracy and precision of both visual and auditory localizations.

Additionally, while previous studies on unisensory biases and variances have enhanced an overall understanding of how the senses process information from the surrounding world, in real-world settings, objects usually produce signals that simultaneously stimulate multiple sensory modalities. In conditions where auditory and visual stimuli co-occur in close spatial proximity, it has been well-documented that auditory localizations are often pulled in the direction of a visual stimulus [18,22–26]. Known as the “Ventriloquist illusion” [27], this effect has been demonstrated in many experiments, lending support to the idea that the most reliable sense in a given dimension exerts a strong influence over other noisier senses as estimates about the world are produced [28].

While many studies have focused on how far this “spatial window” of audiovisual integration extends [29], one important question remains: when visual and auditory stimuli *co-occur* at the *same* spatial location (which is generally the case when an event produces both visual and auditory stimulation), which modality’s bias emerges during localization? Is it the visual bias, the auditory bias, or something else? If, as many studies indicate, visual localization is biased towards the center, and auditory localization is biased towards the periphery, three possibilities exist: vision dominates on most trials, and a central bias emerges; the auditory bias dominates, and a peripheral bias emerges; the biases interact, and a hybrid bias emerges in the bimodal localizations. It has been shown previously that even when the stimuli are congruent in both space and time, they may be perceived to have a common cause or independent causes. Therefore, it is also important to explore whether the nature and presence of bias in multisensory conditions is influenced by the inference of common cause.

Finally, another important question that has not been previously explored is the following: if indeed the visual and/or auditory localization of stimuli in space is biased and inaccurate, what underlies this bias? Is this bias in localization a result of bias in the sensory representation of the space, a result of general a priori expectation of location of events in the world, or the result of a combination of these two possibilities? In the former case, the sensory encoding of the stimuli is compressed towards the center of the visual space, and this early sensory bias leads to a mislocalization of stimuli towards the center without any effect of prior expectations for the location of the stimuli. In the latter case, the sensory representation of the visual space may be perfectly accurate and unbiased, but due to the task demands or expectations about the location of the stimuli (in this task or in general), the localization may be biased towards the center. The third possibility is that the localization bias stems from a combination of both of these mechanisms. Teasing apart these three different scenarios requires quantitative computational modeling of the data, as explained below.

The behavioral findings in this study showed that both visual and auditory localizations were indeed biased, with visual localizations on average biased towards the center and auditory localizations on average biased away from the center. To address whether these biases in spatial perception stem from a bias in sensory representations or from a prior expectation of location, we employed the causal inference model of multisensory perception [30–32] to quantitatively characterize both sensory representations and prior expectations of each individual observer. This model has been very successful in accounting for human observers’ data in a variety of multisensory tasks [30–35] and a recent brain imaging study has provided further support for the brain utilizing this computation in the spatial localization task that is used in this study [36,37]. Importantly, this model can reliably provide a quantitative estimate of several components of perceptual processing for each individual observer.

**Fig 1:**
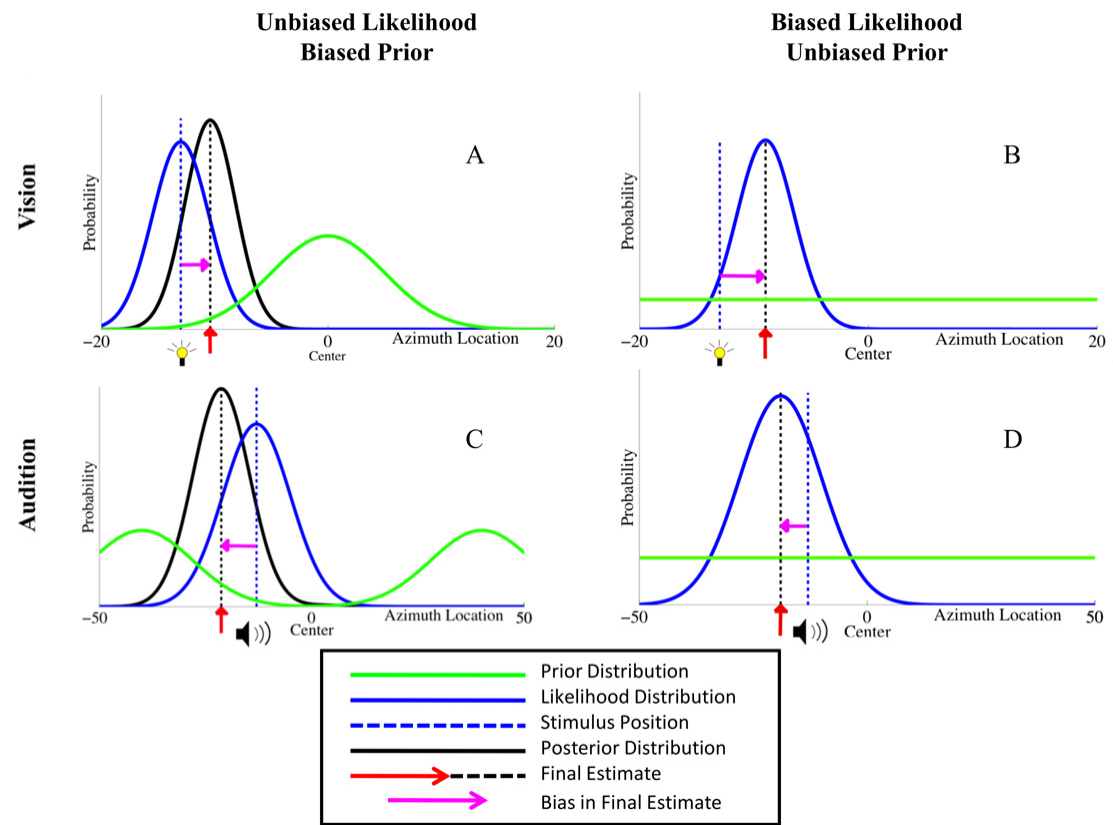
Possible underlying mechanisms for the biases observed in subjects’ localization responses. Zero on the x-axis represents the center. In panels (B) and (D), the likelihood and posterior functions are overlapping and are shown in blue. In the visual domain, (A) a prior distribution located at the center of visual space could draw localization estimates towards the center, or (B) the likelihood distributions themselves could be biased, drawing sensory estimates towards a central location. In the auditory domain, (C) a prior bias for periphery could push perception of peripheral target estimates further away from the center of space, or (D) the likelihood distributions themselves maybe biased away from the center. Various combinations of these computational mechanisms were tested in the different proposed models (see Model Comparisons section).

**Fig 2:**
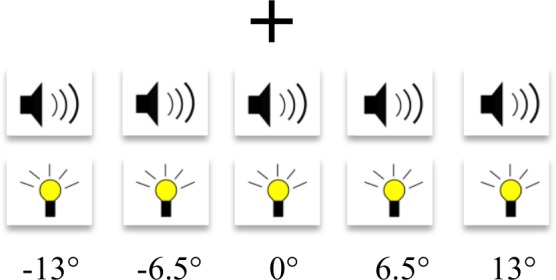
The spatial localization paradigm. Stimuli could be presented from one of five locations, ranging from -13 to +13 degrees.

In a Bayesian framework, the final perceptual estimate is based upon a combination of the sensory representation (i.e. likelihood distribution) and pre-existing expectation (i.e. prior). Fig 1 shows different kinds of underlying mechanisms that could produce biases similar to those shown by most subjects in our localization task. In the visual domain, either (1) an *a priori* bias for center in expectation of location of visual stimuli (Fig 1A), or (2) a bias in the sensory representation of visual stimuli towards the center (the likelihood means shifted towards center with the amount of shift being proportional to the degree of stimulus eccentricity) could account for biased perception (Fig 1B). In the auditory domain, either (1) an a priori bias towards periphery (Fig 1C), or (2) a bias away from the center in sensory representations (Fig 1D; shifts of likelihood means away from the center with the degree of shift proportional to stimulus eccentricity) could account for participants’ behavior. The exhibited behavioral biases may also be due to a combination of biased likelihood and biased priors. To investigate which of these options is indeed the mechanism underlying the observed biases, we implemented all three types of mechanisms (biased likelihoods, bias priors, a combination of the two) into a Bayesian Causal Inference model and performed quantitative model comparisons to determine which computational mechanism best accounts for the behavioral data.

All previous models of multisensory spatial localization assume that sensory representations are unbiased, and that the main benefit in integrating stimuli is improvement in the overall *precision* of the combined estimate. For example, maximum likelihood estimation models have proposed that auditory and visual signals can be represented by distributions centered at the true location of each stimulus, with the combined audiovisual estimate exhibiting a smaller variance than the unisensory estimates [38–40]. The reduced variance of the combined estimate has been considered to be the main reason why it may be advantageous for the brain to integrate redundant sensory information in a complex world [41,42]. Here, we explore the effect of integration on the *bias* of the estimates and examine whether localization estimates become less biased, more biased or remain the same as a result of integration as compared to unisensory conditions.

In order to address all of these questions, we analyzed psychophysical data and quantitatively characterized the perceptual components involved in the spatial perceptual process using computational modeling. Specifically, the study had five aims: (1) to quantify unisensory spatial biases and variances in the visual and auditory modalities using a large dataset, (2) to investigate how biases are reconciled when subjects localize spatially congruent bisensory stimuli, (3) to determine whether the biases that emerge in spatially congruent bisensory trials depend on an observer’s inference about a common cause, (4) to determine whether the biases in spatial localization are due to a bias in the sensory representations, prior expectations, or both, and (5) to examine how the biases in bisensory conditions compare with biases in unisensory conditions.

## Materials and Methods

### Ethics Statement

This study was conducted according to the principles expressed in the Declaration of Helsinki. Each subject also signed a consent form approved by the UCLA IRB.

### Participants

A total of 412 subjects (ages 18–55) participated in our experiment; since our measurement of localization biases are means that can be influenced by extreme outliers, 28 subjects were excluded because their responses for each of the five unisensory conditions in either the visual or auditory modalities were more than three times the inter-quartile standard deviation for the tested location. This exclusion criterion ensured that we would avoid analyzing data that may have been due to sloppiness or negligence with the response device. All participants verbally reported that they did not have a history of any neurological conditions (seizures, epilepsy, stroke), had normal or corrected-to-normal vision and hearing, and had not experienced head trauma.

### Apparatus and Stimuli

Eligible participants sat at a desk in a dimly lit room with their chins positioned on a chinrest 52cm from a projection screen. The screen was a black, acoustically transparent cloth subtending much of the visual field (134° width ° x 60° height). Behind the screen were 5 free-field speakers (5 x 8 cm, extended range paper cone), that differed in azimuth by 6.5° and were placed 7° below fixation. The middle speaker was positioned directly below the fixation point, and two speakers were positioned to the right and two to the left of fixation. The visual stimuli were presented overhead from a ceiling mounted projector set to a resolution of 1280 x 1024 pixels with a refresh rate of 75 Hz.

Prior to the presentation of any stimuli in the experiment, participants were required to have their gaze centered on the central fixation point. To ensure that participants’ gaze for each trial was starting from the same location, gaze position and fixation time were recorded at 60Hz with a ViewPoint eye tracker (Arrington Research, Scottsdale, AZ) fixed to the chinrest and PC-60 software (version 2.8.5,000). Stimuli were not displayed until the recorded gaze angle was within 3.0° of the fixation point and the fixation time was greater than 250 ms. Viewing of the stimuli was binocular, although only movements of the right eye were tracked. The eye tracker was adjusted for each participant before the test session to ensure that the entire eye was being monitored, and a calibration task was performed before trials for the experiment began. A separate computer controlled presentation of stimuli and recorded behavioral responses using MATLAB (version 7.6.0, R2008a). A wireless mouse was used to record the behavioral responses.

The visual stimuli used in the experiments were white disks (.41 cd/m2) with a Gaussian envelope of 1.5° FWHM, presented 7° below the fixation point on a black background (.07cd/m2), for 35 ms. The center of visual stimuli overlapped the center of one of the five speakers behind the screen positioned at -13°, -6.5°, 0°, 6.5°, and 13°. Auditory stimuli were ramped white noise bursts of 35 ms measuring 59 dB(A) sound pressure level at a distance of 52 cm. The speaker locations were unknown to all of the participants in the experiment.

### Procedure

Participants began each session with 10 practice trials requiring localization of unisensory auditory stimuli. This practice session ensured that participants were using the mouse properly, understood the instructions, and were fulfilling the fixation requirements for each trial. Each trial started with the fixation cross, followed after 750 ms (if the subject was fixating properly) by the presentation of stimuli. 450 ms after the stimuli, fixation was removed and a cursor appeared on the screen vertically just above the horizontal line where the stimuli were presented at a random horizontal location in order to minimize response bias. Following the removal of fixation, the scene was entirely dark except for the cursor. The cursor was controlled by the trackball mouse placed in front of the subject, and could only be moved in the horizontal direction. Participants were instructed to “move the cursor as quickly and accurately as possible to the exact location of the stimulus and click the mouse.” This enabled the capture of continuous responses with a resolution of 0.1 degree/pixel. No feedback about the correctness of responses was given. Participants were allowed to move their eyes as they made their response, so the fixation requirement was dropped following the presentation of the stimuli.

Following the brief practice session, participants began the localization session, which consisted of 525 trials of interleaved auditory, visual, and audiovisual stimuli presented in pseudorandom order. The stimulus conditions included 5 unisensory auditory locations, 5 unisensory visual locations, and 25 combinations of auditory and visual locations (bisensory conditions), for a total of 35 stimulus conditions, shown in Fig 2 below. Fifteen trials of each of the 35 conditions were presented in pseudorandom order, lasting about 45 minutes, including breaks.

### Bayesian Causal Inference Model

The Bayesian causal inference model has been shown in multiple studies to account for the human multisensory spatial perception very well [30–33] and it has been shown to outperform other proposed models of multisensory spatial perception [30]. Moreover, a human neuroimaging study recently provided further support for the Bayesian Causal Inference being carried out in human brain in the spatial localization task, using the same task used in current study [37]. Therefore, we used this model to investigate the characteristics and perceptual components of human spatial processing in our localization task. In previous formulations of this model it had been assumed that (1) the likelihood distributions of sensory stimuli are unbiased (centered at the true locations of stimuli in the world), (2) visual and auditory localizations are both impacted by a meta-modal central prior over space, and (3) the variance of sensory representations (likelihood functions) is uniform across all eccentricities. Considering our behavioral results, which reveal non-zero biases of differing direction and magnitude in the visual and auditory modalities, it appears that these simplifying assumptions are not quite warranted, and the model would need to be enhanced to allow flexibility in the representation of likelihoods and/or priors.

The nervous system does not have access to the true events *s* in the world. Instead, it only has access to the noisy sensory representations, *x*_*v*_ *and x*_*a*_ (denoting visual and auditory sensations, respectively). Taking into account both the noisy sensory representations (likelihoods) and prior knowledge of the world (priors), the brain makes an inference about whether the sensory signals come from the same source (*C* = 1) and should be integrated or the signals come from different sources (*C* = 2) and should be segregated (see [30] for an image of the graphical model).

Thus, the posterior probability of an event *s* is conditioned on the causal structure of the stimuli (C = 1 or C = 2):

$$p(s|x_{A},x_{V}\;;\;C=1)=\frac{p(x_{A}|s)p(x_{V}|s)p(s)}{p(x_{A},x_{V})}$$ (1)

$$p(s_{A}|x_{A}\;;\;C=2)=\frac{p(x_{A}|s_{A})p(s_{A})}{p(x_{A})}$$ (2)

$$p(s_{V}|x_{V}\;;\;C=2)=\frac{p(x_{V}|s_{V})p(s_{V})}{p(x_{V})}$$ (3)

The estimate of the location of the stimuli will be based on these posterior probabilities. However, since the causal scenario is not known by the nervous system, this also must be inferred based on the available sensory evidence and prior information. This can also be computed using Bayes’ Rule:

$$p(C|x_{A},x_{V})=\frac{p(x_{A},x_{V}|C)p(C)}{p(x_{A},x_{V})}$$ (4)

Therefore,

$$p(C=1|x_{A},x_{v})=\frac{p(x_{A},x_{V}|C=1)p_{c}}{p(x_{A},x_{V}|C=1)p_{c}+p(x_{A},x_{V}|C=2)(1-p_{c})}$$ (5)

where P_c_ is the prior probability of a common cause, and the likelihood terms can be computed by integrating over the latent variable *s*:

$$p(x_{A},x_{V}|C=1)=\int \;p(x_{A}|s)p(x_{V}|s)p(s)ds$$ (6)

$$p(x_{A},x_{V}|C=2)=\int \;p(x_{A}|s_{A})p(s_{A})ds_{A}\cdot \int \;p(x_{V}|s_{V})p(s_{V})ds_{V}$$ (7)

The posterior probability of independent causes is computed as follows:

$$p(C=2|x_{A},x_{V})=1-p(C=1|x_{A},x_{V})$$ (8)

Having calculated the probabilities of each causal structure, and having calculated the optimal estimates for spatial localization under each causal structure, we now need to obtain estimates given *uncertainty* about the causal structure. Previously it has been shown [24] that the vast majority of observers use a probability matching strategy as follows:

$$\begin{array}{l} {\hat{s}}_{A}=\left\{ \begin{array}{l} {\hat{s}}_{A,\;C=1}\;if\;p(C=1|x_{A},x_{V})\;>\xi \\ where\;\xi \in [0\;:\;1]\;uniform\;distribution\\ {\hat{s}}_{A,\;C=2}\;if\;p(C=1|x_{A},x_{V})\leq \xi \\ and\;sampled\;on\;each\;trial \end{array}\right.\\ {\hat{s}}_{V}=\left\{ \begin{array}{l} {\hat{s}}_{V,\;C=1}\;if\;p(C=1|x_{A},x_{V})>\xi \\ where\;\xi \in [0\;:\;1]\;uniform\;distribution\\ {\hat{s}}_{V,\;C=2}\;if\;p(C=1|x_{A},x_{V})\leq \xi \\ and\;sampled\;on\;each\;trial \end{array}\right. \end{array}$$ (9)

Therefore, here we used this strategy to model the observers’ data. We used a generative model to simulate 10,000 *x*_*A*_’s and *x*_*V*_’s for each experiment condition (35 total) using the preceding equations. Trial-to-trial variability is introduced by sampling the likelihoods from Eqs 1–3 from a normal distribution centered at the true sensory location, plus a bias term that scales linearly with eccentricity of the stimulus. Thus, if subjects’ sensory representations are not centered at the true locations, this bias term could potentially reflect this systematic shift in the likelihoods. The variability in the normal distribution from which the likelihoods are sampled scales with eccentricity of the stimulus. The prior described in the above equations changes depending on the model version, and can either be the biased central prior, the biased peripheral prior, or both, (see Fig 1).

We quantitatively compared six models to determine which model could best account for participants’ data. Different mechanisms were incorporated in the different models, resulting in different combinations of free parameters. All of these parameters are shown in Table 1, with a brief description of their corresponding mechanism.

**Table 1:** Glossary table of all potential parameters.

Parameter	Description
P_c_	The prior probability of integrating visual and auditory signals; the tendency to bind
σ_V_	Standard deviation of the visual likelihood; the noise in the visual sensory representation
σ_A_	Standard deviation of the auditory likelihood; noise in the auditory sensory representation
Δx_V_	The shift in the center of the visual likelihood; the bias in the visual representation
Δx_A_	The shift in the center of the auditory likelihood; the bias in the auditory representation
Δσ_V_	The increase in visual likelihood std dev with each increase in eccentric position
σ_P_	Standard deviation of the metamodal spatial prior
x_P_	The mean of the metamodal spatial prior
σ_VP_	Standard deviation of the visual spatial prior
x_VP_	The mean of the visual spatial prior
σ_AP_	Standard deviation of the auditory spatial prior
x_AP_	The mean(s) of the auditory spatial prior

Three parameters were included in every model: the prior probability of a common cause (P_c_), the standard deviation of the visual likelihood (σ_V_), and the standard deviation of the auditory likelihood (σ_A_). Two models incorporated the biased likelihood mechanisms, including a symmetric shift in the visual likelihood mean that increases with eccentricity (Δx_V_), a symmetric shift in the auditory likelihood mean that increases with eccentricity (Δx_A_); one of these models included a change in the visual likelihood variance which increases with eccentricity (Δσ_V_), while the other did not. Two models incorporated the biased prior mechanisms, including a visual central prior to capture a central bias (σ_VP,_ x_VP_) and a peripheral spatial prior to capture a peripheral auditory bias (with the mean and variance, σ_AP,_ x_AP,_ reflecting the symmetric aspects of each part of the bimodal distribution); one of these included an additional metamodal spatial prior, while the other did not. We also included the original model [31], which does not include either the biased likelihood nor the biased prior mechanisms, as the baseline for comparison. Finally, we included a hybrid model that incorporated all biased likelihood mechanisms (Δx_V_, Δx_A_, Δσ_V_) as well as a metamodal spatial prior. These models are summarized in the results section. Since the different models have different numbers of free parameters, in addition to reporting their model fits (in log likelihood) we also report the BIC value of each model, which is a measure of goodness of fit that penalizes models with larger number of free parameters. The six models described above were applied to the data from each of the 384 observers, and log likelihood and BIC values were computed for each model’s fit to each observer.

## Results

The trials in which only a visual stimulus or only an auditory stimulus was presented were analyzed for characterizing localization in “unisensory” conditions. The trials in which both visual and auditory stimuli were presented and occurred in the same location (congruent audiovisual trials) were used to characterize the localization behavior in the “bisensory” condition.

### Accuracy of Localization in Unisensory Conditions

**Fig 3:**
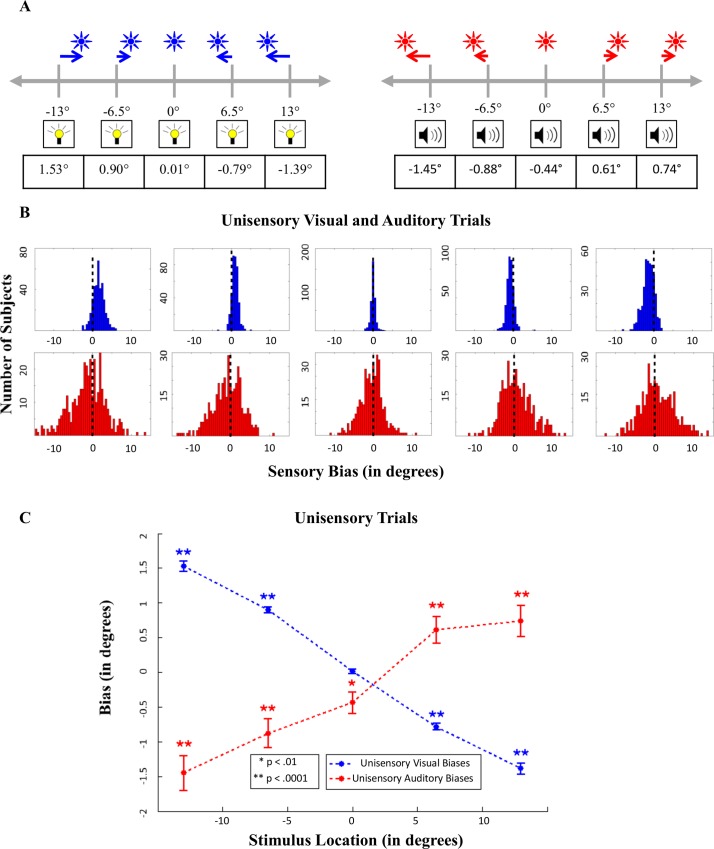
Biases present in localizations on unisensory trials. A) Average biases across subjects. B) The distribution of subjects’ individual biases (based on each subjects’ mean across 15 trials) for each of the five locations, from -13 degrees in the leftmost column, to +13 degrees in the rightmost column. Visual biases are shown in blue, and auditory biases are shown in red. C) Positive numbers indicate rightward biases, and negative numbers indicate leftward biases. SEM bars (computed over all subjects) are shown around the mean bias in each modality for each location.

For each observer, the average error in localization (across 15 trials) in the unisensory trials was calculated for each spatial position and for each modality. The mean and distribution of these biases across observers are shown in Fig 3A, 3C and 3B, respectively. Participants’ average localizations for unisensory visual stimuli exhibited a bias for localizing peripheral stimuli closer to the center of visual space. Analysis of unisensory-visual trials showed that 63% of subjects (242) exhibited central biases for all four peripheral locations of visual stimuli, and 84% of subjects (319) exhibited central biases for three out of the four peripheral locations. This trend for a central bias tended to increase as the eccentricity of the visual stimulus increased. In the auditory domain, subjects’ average localizations for each location revealed the opposite trend: average localizations for eccentric stimuli exhibited a peripheral bias for localizing the stimuli (Fig 3A and 3C). However, further analysis revealed heterogeneity among subjects for this trend: 25% of the subjects exhibited consistent peripheral biases for all four eccentric locations, but 15% of participants exhibited consistent central biases. Overall, though, 45% of the participants exhibited a peripheral bias for three out of the four locations, indicating that a substantial proportion of subjects localized eccentric auditory stimuli further away from the true locations of the sounds.

Bonferonni-corrected one-sample t-tests for all peripheral localizations in both modalities were all highly significant (p < .0001), indicating the responses in both the visual and auditory modalities for peripheral locations were biased away from the true location of the stimulus. For the central (0°) localizations, there was no bias in the visual modality (t(383) = 1.5, p > .05), but a small bias to the left for the auditory modality was observed (t(383) = -2.9, p = .0042). Previous research has indicated that participants often display an auditory localization bias contralateral to the preferred hand [43], so this slight bias could be explained by the near certain-assumption that the majority of our participants were right-handed.

Finally, as can be seen in Fig 3B, the distributions of biases in both the auditory and visual modalities are unimodal, indicating that despite some variability in biases across subjects, there do not appear to be distinct subgroups (i.e. “central-bias” subjects and “peripheral-bias” subjects) that are easily classified. Importantly, the distributions in biases shown in this figure reveal the value of acquiring large sample sizes when investigating spatial perception: reporting averages from small samples could not only reflect skewed trends based on the influence of outliers, but also obscure important variability across subjects in terms of the extant biases. Especially in the auditory domain, studies with smaller sample sizes have not previously revealed this amount of heterogeneity in biases in auditory space [14,16].

### Accuracy of Localization in Bisensory Conditions

**Fig 4:**
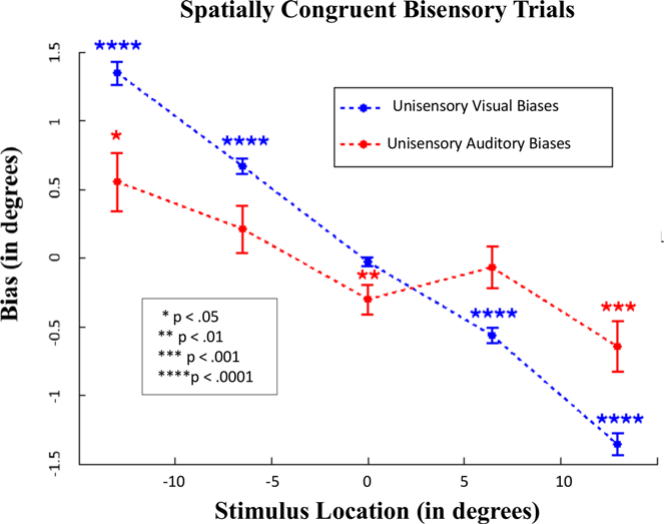
Visual and auditory biases on spatially congruent bisensory trials. First, each subject’s average bias (over 15 trials) was computed for each of the five locations. Then, the mean across 384 subjects was calculated. Error bars represent standard error of the mean across subjects’ averages.

**Fig 5:**
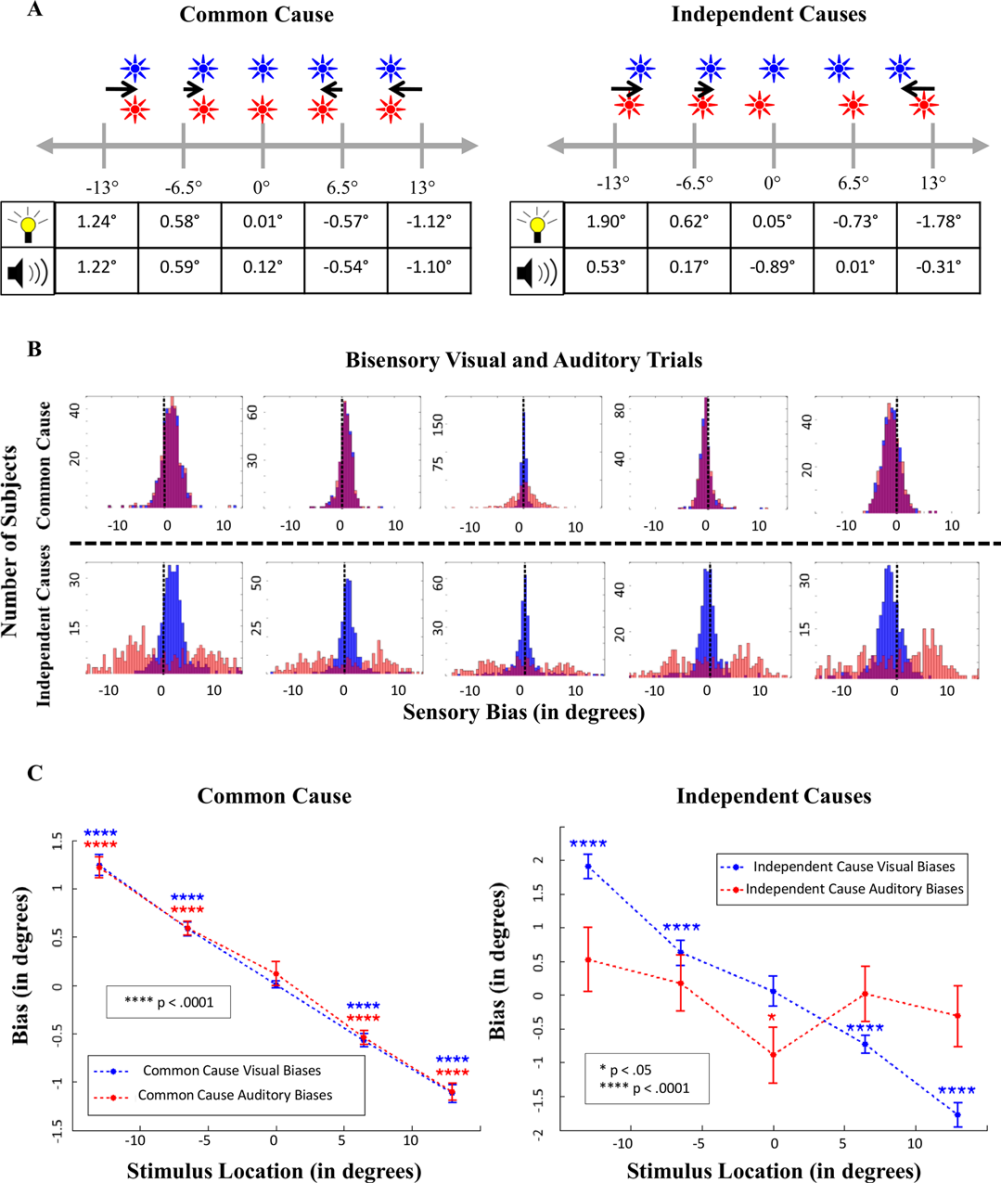
Bisensory trials, classified by inferring one common cause, or two independent causes. Panel A shows average biases and panel B displays the distribution of subjects’ individual biases for each location. In Panel C, positive numbers indicate rightward biases, and negative numbers indicate leftward biases. SEM bars are shown around the mean bias in each modality for each location.

Given that the average biases in the visual and auditory modalities are in opposite directions, an intriguing question arises: in which direction is this conflict resolved when auditory and visual stimuli co-occur at the same location? Analysis of congruent bisensory trials (i.e., trials on which the visual and auditory stimulus co-occurred at the same spatial location) revealed that on average, both the visual and auditory modality exhibited *central* biases (Fig 4). However, as shown below (see Fig 5), further analysis of these trials revealed that the bias that emerged in the auditory modality was dependent on whether the observer inferred a common cause or distinct causes for the stimuli.

Previous research investigating auditory localizations has shown that the bias that emerges in the auditory domain is contingent upon whether observers perceive a simultaneous visual stimulus as unified with or independent from the auditory stimulus (see Fig 5 in [26]). Observers’ explicit reports indicate that even with spatially and temporally congruent flashes and sound bursts, sometimes a common cause is perceived, but at other times discrepant causes are perceived, and this inference can affect the amount of bias that emerges during the localization task. This phenomenon has been explained quantitatively by a Bayesian causal inference model [30]. Furthermore, recalibration of auditory space by vision has been shown to strongly depend on whether or not a common cause is perceived for the two stimuli [44].

Therefore, we investigated whether the inference of a common cause influences the bias in bisensory conditions. As in [44], we classified the congruent bisensory trials into “common-cause” and “independent-cause” trials based on the observer’s responses. If the visual and auditory localizations were within two degrees of each other, we considered the trial as a common-cause trial (as the stimuli are perceived to have the same location, thus reflecting the inference of a common cause). If the visual and auditory localizations were more than five degrees apart, we considered the trial to be generated by independent causes (as this degree of discrepancy between the two percepts is inconsistent with the inference of a common cause, and is unlikely to be due to motor error). Trials in which the discrepancy between visual and auditory responses were in between 2 and 5 degrees were excluded from analysis (due to uncertainty about the inference of causal structure). The results are shown in Fig 5.

As can be seen in Fig 5C, on “common cause” trials both modalities show a considerable central bias, which increases as eccentricity of the target increases. Interestingly, comparing these common cause trials with the corresponding unisensory trials in each modality reveals an important effect: when trials are integrated, the amount of bias *decreases* in each modality, and the localizations become closer to the veridical location of the stimulus (p < .005 for t-tests for all eccentric locations). For example, in the visual modality, unisensory localizations at the -13 degree location exhibited a bias towards the center of 1.53 degrees, but when visual localizations are integrated with an auditory stimulus at this location, the bias decreases to 1.24 degrees. This reveals an important new finding: multisensory integration not only increases precision in the final spatial estimates of stimuli (which was previously well-established), but also increases the *accuracy* of the estimates by reducing biases and bringing the final estimates closer to the true stimulus location.

Localizations on trials where independent causes were inferred are much more variable: while the visual modality shows a central bias that appears to increase as eccentricity increases, the bias that emerges in the auditory modality is irregular, and the trend less clear. This variability is apparent in Fig 5C, where the irregularity in auditory localizations on trials where independent causes were inferred is evident.

Thus, to summarize: in spatially congruent audiovisual trials in which a common cause is perceived, the visual bias dominates, however, the degree of bias is smaller relative to unisensory trials. In contrast, on segregated trials, while the visual modality still exhibits a central bias, auditory biases are extremely variable.

### Precision of Localization

**Fig 6:**
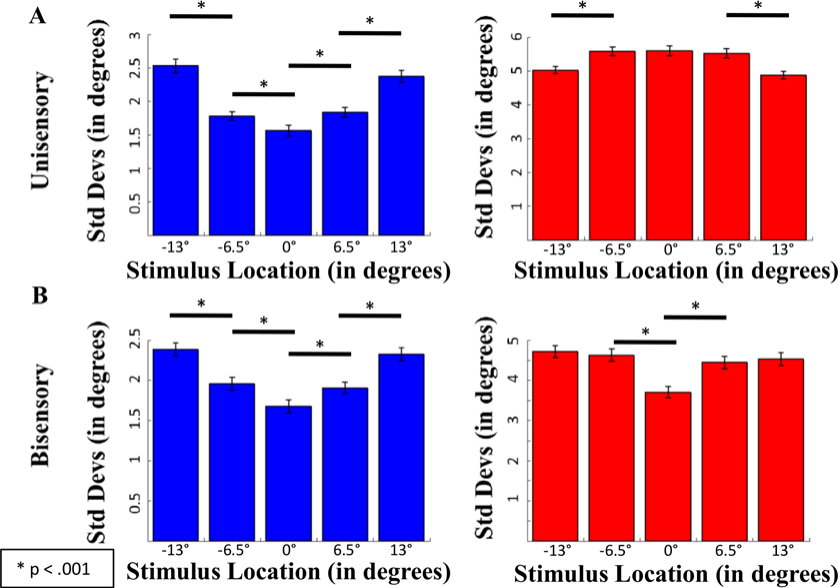
Standard deviations for all five stimulus positions. (A) Unisensory visual (blue) and unisensory auditory (red) localizations, and (B) bisensory visual (blue) and bisensory auditory (red) localizations are shown for each modality.

Analysis of the variability in unisensory trials (Fig 6) showed the following: the standard deviation of visual localizations was not equivalent across conditions (F(4,1532) = 39.467, p < .001), and increased as the eccentricity of the stimulus increased (p < .001 for all paired-samples t-tests between adjacent conditions). The standard deviation of auditory localizations also revealed differences contingent on stimulus eccentricity (F(4, 1532) = 16.32, p < .001), but in a different way. That is to say, variability in the two auditory standard deviations surrounding the zero-degree location (+6.5 and -6.5) degrees) were not significantly different from the variability at the central location (p > .05 for both paired-samples t-tests), but variability in the peripheral locations (-13 and +13 degrees) were significantly different from the variability in localizations at the spatially adjacent neighbors (p < .001 for both paired-samples t-tests).

We also evaluated the precision in visual and auditory localizations on trials where the visual and auditory stimuli co-occurred at the same locations in space. In the visual modality, similar to the unisensory trials, the standard deviations for visual localizations increased as eccentricity increased (F(4,1532) = 29.89, p < .001)). In the auditory modality, although a significant effect emerged (F(4,1532) = 22.73, p < .001), none of the peripheral locations were significantly different from one another, and the only significant difference was that variance in peripheral locations was larger than that of the center (p < .001 for all paired-samples t-tests).

### Model Comparisons

Table 2 shows the results of model fits for each of the 6 models. The first two columns describe each model, columns 3 and 4 show the log likelihood and BIC values averaged across all observers, column 5 shows the number of subjects best fit by each model, and column 6 shows the average R^2^ values [45] across all observers. As can be seen, the 8-parameter model accounts for the observers’ data the best, as it has the best-fitting average log likelihood value, the lowest average BIC value (i.e. the best account of the data even after penalizing it for having larger number of free parameters than some of the other models), the largest R^2^ value, and the highest number of subjects that are best fit by its iteration. In fact, the number of participants whose data is best accounted for by the 8-parameter model is one order of magnitude larger than the runner up model, providing compelling evidence for the superiority of this model over the other models in explaining the data.

**Table 2:** Summary of modeling results.

Model Description	Model Parameters	Log Likelihood	BIC	# of Subjects Best Fit	R^2^
4-Param Original Model: Meta-modal Central Bias prior	Pc, σ_V_, σ_A,_ σ_P_	-2416.0	4857.1	2	.807
5-Param Likelihood Model: Shift in Likelihood Means	Pc, σ_V_, σ_A,_ Δx_V,_ Δx_A_	-2357.9	4747.2	0	.827
6-Param Likelihood Model: Shift in Likelihood Means, Variable Visual Likelihood Variance	Pc, σ_V_, σ_A,_ Δx_V,_ Δx_A,_ Δσ_V_	-2333.9	4705.3	33	.831
**8-Param Hybrid Model**: **Shift in Likelihood Means, Variable Visual Likelihood Variance, Meta-Modal Central Bias Prior**	**Pc, σ**_**V**_ **, σ**_**A,**_ **Δx**_**V,**_ **Δx**_**A,**_ **Δσ**_**V,**_ **σ**_**P,**_ **x**_**P**_	**-2311.8**	**4673.7**	**290**	**.842**
7-Param Prior Model: Visual Central Prior & Auditory Peripheral Prior	Pc, σ_V_, σ_A,_ σ_VP,_ x_VP,_ σ_AP,_ x_AP_	-2352.3	4748.4	21	.827
9-Param Prior Model: Visual Central Prior, Auditory Peripheral Prior, & General Spatial Prior	Pc, σ_V_, σ_A,_ σ_VP,_ x_VP,_ σ_AP,_ x_AP,_ σ_P,_ x_P_	-2351.7	4765.7	38	.828

A one-way repeated-measures ANOVA applied to the BIC values [46] showed that the model fits were significantly different from one another (F(5, 1915) = 248.1, p < .001). Furthermore, a post-hoc t-test between the two best models (the 6-parameter and 8-parameter model) showed that the 8-parameter model was significantly superior (t(383) = 10.2, p = .001). Thus, a model that incorporates biases in the sensory representations and a general (metamodal) prior bias for center best captures observers’ performance in our multisensory localization task.

**Fig 7:**
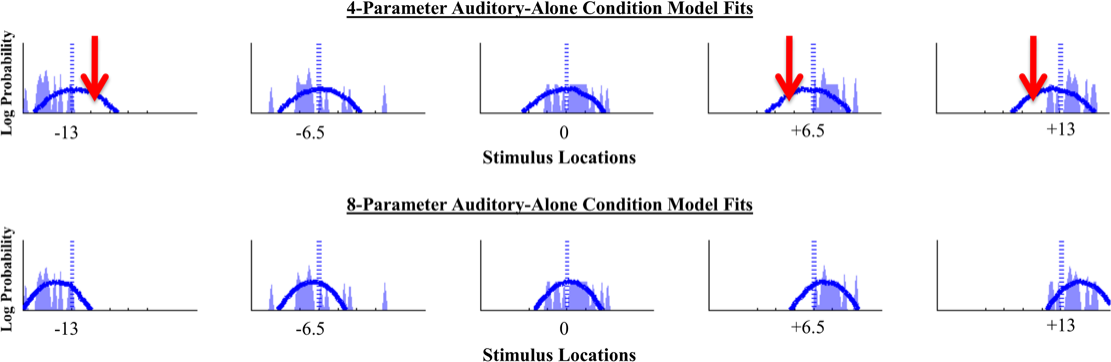
Auditory-alone condition model fits for one randomly selected subject. Plotted across the columns are model fits for each of the five auditory locations, from -13 to +13. The dotted light blue line shows the true stimulus location, the subject’s data is plotted in the shaded light blue regions, and the solid dark blue line shows the model’s fit to the data. As shown by the red arrows, simple models that assume unbiased sensory representations make considerable errors in estimating subjects’ response, as simulated response distributions are centered near the true stimulus locations. By allowing the sensory representations to vary, biases can be more fully accounted for, as is shown in the fits for the 8-parameter model in the second row.

Previous versions of the model have fit data quite well (as also evidenced in the high R^2^ values for all model fits) [30,31]. It is therefore important to note that simpler versions of the model in this study did not do an inadequate job of fitting the overall data; instead, they were limited primarily in their abilities to account for the unisensory data, which revealed sensory biases in eccentric positions. Shown in Fig 7 are model fits for the auditory-only conditions from one randomly selected subject. When plotted, it becomes clear that the simple 4-parameter model fails to account for peripheral auditory biases, and thus more complex models are needed to fully account for the phenomenon of sensory biases.

In the winning hybrid model (8 parameters), the optimized value of the mean for the general meta-modal prior was almost zero, reaffirming the central bias that had been assumed in the original 4-parameter model [30,31]. Therefore, the only difference between the winning model and the original 4-parameter model is in the bias in visual and auditory sensory representations, and the increase in visual likelihood variance as a function of eccentricity.

## Discussion

### Auditory and Visual Perception of Space Is Indeed Biased

Many previous studies have suggested that humans’ visual and auditory localizations in surrounding space are biased and inaccurate [1,2,13,15]. If such biases are truly present in human perception of space, they would pose a riddle and warrant further investigation into their characteristics and function. However, previous studies have been based on relatively small sample sizes that render the results prone to outliers and other statistical irregularities. To examine whether these biases are real (or reflect noise or other phenomena), we used a very large sample size (384 subjects) and an experimental design minimizing the potential for induction of any perceptual or response biases. The experiment consisted of a large variety of unisensory and bisensory stimulus conditions presented in pseudo-random order with a uniform distribution across space, and a fairly large number of trials per condition. Furthermore, the positions of the auditory and visual sources were unknown to the observers, no feedback was provided, and the initial position of the response cursor was randomized. Thus, the experimental design could not induce any perceptual or response bias for any given position in space. By using a rigorous experimental design and acquiring a large dataset, we aimed to obtain a more definitive answer to the question of the existence and nature of any biases in the visual and auditory perception of space.

The results demonstrated that when observers were asked to localize briefly presented visual and auditory targets that differ in azimuth, on average, localizations of unisensory visual targets displayed a central bias that increased with eccentricity, and localizations of unisensory auditory targets displayed a peripheral bias that increased with eccentricity. Our finding of a central bias in visual localization is consistent with most studies in this area, especially those in which the cursor was visible during response, which thus minimizes motor error [1,6,13,15]. While the vast majority of observers in our sample showed a clear and consistent bias towards center in vision, the biases exhibited by observers in the auditory modality were more variable and less consistent, but still on average displayed a peripheral bias. This finding is also consistent with those of several previous studies of auditory spatial localizations [6,13,14].

Localizations for spatially congruent bisensory stimuli exhibited biases that were contingent upon the subject’s inference of the causal structure of stimuli: when a common cause was inferred, the visual bias dominated. When independent causes were inferred, vision continued to exhibit a bias towards the center of space, but the auditory modality on average showed a peripheral bias (as in the unisensory auditory condition).

These findings are quite consistent with research requiring explicit judgments of unity or discrepancy [26], indicating that sensory systems implicitly perform inference processes, and that the biases that emerge are contingent upon whether or not sensory signals were integrated or segregated. Interestingly, on trials for which a common cause was perceived, the opposing biases were reconciled in the following manner: the visual bias dominated, although its magnitude was reduced. This finding strongly suggests that the observed biases in the unisensory conditions stem from a bias in sensory representations. Because visual representations are significantly more precise (and reliable) than the auditory representations, in bisensory conditions the visual signals dominate, and therefore, the visual bias dominates the auditory bias. However, in principle, any bias in perception could originate from either a bias in sensory representations (modeled by likelihood functions) or a bias in the expectations or model of the world (prior).

### The Biases in Localization Largely Reflect Biases in Sensory Representations

While the findings discussed above suggest that biases in bisensory conditions are more consistent with a bias in the sensory representations, to address this question rigorously, one needs to quantitatively model these scenarios and perform model comparisons to determine which mechanism(s) account for the behavioral data the best.

To do this, we used the Bayesian Causal Inference model that has been shown to account for human observers’ multisensory perception quite effectively [30–35,37]. We compared models incorporating a bias in likelihoods to those incorporating a bias in prior expectations, and also tested one model that incorporated both mechanisms. We fitted the model parameters to each observer’s data, and compared the models based on data from 384 observers. Consistent with the qualitative observation discussed above, the quantitative model comparison results revealed that indeed a bias in auditory and visual spatial sensory representations is necessary to account for the participants’ data. But over and above these sensory biases, a general (metamodal) prior bias for center appears to also exist in perception of space.

### Why Do Biases Exist?

Why should the visual system be biased in perceiving locations towards the center? Upon first glance, this would appear to be a tremendously suboptimal strategy, but insights from phenomena in other perceptual domains may provide some possible explanations. For instance, saccadic undershooting for eye movements to peripheral stimuli has been demonstrated conclusively and would seem to be a suboptimal strategy [47,48], but one potential explanation that has been posited is that it represents an advantageous trade-off between accuracy and the movement time needed to reasonably localize the stimulus [49]. Here, we find spatial localization biases in sensory representations. If visual sensory representations were built *only* for this task, then biases could be suboptimal. But sensory representations are calibrated to be optimal for acting in a complex and changing world in a variety of tasks and conditions, and thus, biases in the visual and auditory systems could be optimal if they are due to an advantageous trade-off; for example, between foveal acuity and peripheral accuracy. Due to the much larger number of neurons representing the fovea compared to peripheral regions [50], the representation of space may be skewed in that direction if spatial representation involves a population code [51]. It seems possible that this cortical magnification effect may skew visual localizations towards the center.

One recent study investigating the perception of visual orientation and spatial frequency posits another possible account: for a Bayesian observer, perceptual priors match stimulus distributions in the world, and sensory likelihoods (i.e. sensory representations) become distorted through a process of “efficient encoding” [52]. In this framework, likelihood functions are constrained by the Fisher information in the encoding process, and the Fisher information matches the stimulus distribution in the world. Thus, rather than positing that biased perception is driven entirely by priors (as many previous models have proposed), this study shows that *sensory representations* can themselves be biased, and this approach can account for biases in perceptual behavior that previously appeared to be “anti-Bayesian.” Here, in our study, we similarly find perceptual biases best characterized by biases in sensory representations. Future work should investigate whether behaviors such as orienting to sources of sensory stimulation (objects and events) may produce a disproportional amount of visual stimuli being located in the center and in turn result in the representation of visual space to become biased accordingly.

Additionally, based on everyday subjective experience, visual biases would seem to be quite counterintuitive, as the surrounding spatial world does not appear to be compressed. However, as noted by Rahnev et al. [53], while subjective perception of the periphery *appears* to be accurate and colorful, studies have demonstrated that the visual periphery has proportionally less processing resolution, likely impacting capacities for form and motion perception [54], and color sensitivity [55]. Therefore, subjective assessments of perceptual capacities in the periphery are inaccurate, and comprehensive investigations are needed to quantify the relative impairments in perception outside the fovea. Similar to lack of subjective awareness of color deficiencies in the periphery, here, in the spatial domain, the observers seem to be unaware of their bias in spatial localization.

It should also be noted that this study is not the first to document the counterintuitive phenomenon of distortions in perception of surrounding visual space. For instance, inaccuracies in visual estimates of both egocentric distance estimates and objects’ shapes have been well documented [56]. Subsequently, Bingham et al. have raised the question of how accurate movements are possible despite these visual distortions [57], and indeed, many studies report that perceptual responses and visually-guided reaching actions are dissociated or even uncorrelated [58–61]. As these authors note, it appears likely that feedback plays an important role in minimizing the impact these distortions can have on our movement accuracy, and it also seems that (as we report here between the auditory and visual modalities) multisensory integration can assist in reducing biases present in a given pair of sensory modalities [57].

The findings regarding peripheral auditory biases also raise the question of why these biases should exist in the auditory domain. As noted by [15], prolonged fixation in an eccentric direction shifts the entire map of auditory space in the direction of the fixation point, even for auditory stimuli that are at the same location as fixation. While auditory localizations still exhibit a slight peripheral bias when central fixation is maintained, it appears that when the eyes are allowed to move, auditory space is shifted in the direction of the eye movement. Further support for this idea comes from a study which included one condition where the eyes were allowed to move when localizing the target, and another condition where central fixation was enforced [16]. Results indicated that enforcing fixation greatly *reduced* the magnitude of auditory biases, suggesting that auditory biases may be driven in part by eye movements. Thus, in our task, as eye position was not constrained, the auditory bias may be at least partially due to this mechanism.

Moreover, much is still unknown about how auditory spatial perception is encoded in the human brain. One current idea is that rather than having explicit auditory spatial *maps*, some form of a distributed population code is built by neurons that may have broad tuning curves [62]. As noted in a recent review [63], this population rate code may be able to be characterized as a “hemifield code,” where the firing rates of the relevant auditory neuron population are highest for stimuli in the periphery (-90 and +90 degrees) that maximize the ITD and ILD information available. This idea has substantial support from experimental findings indicating that sound source locations could be encoded by the relative firing rates of the right-tuned or left-tuned auditory cortical neurons [64,65] and has been further supported by recent neuroimaging work [66,67]. Thus, it seems possible that when peripheral stimuli are presented, enhanced activity in the auditory population code could skew perception of auditory space towards eccentric locations. Future research will need to determine exactly how activity in both cortical and subcortical regions contributes to humans’ perception of auditory space.

### Multisensory Integration Reduces the Bias in Spatial Perception

Multisensory integration may be one of the strategies the nervous system attempts to reduce the problem of unisensory biases. Our behavioral data showed that biases in spatial perception were much smaller in bisensory congruent trials (in which both sound and light were presented at the same location), relative to either the unisensory visual or unisensory auditory trials. Therefore, it appears that multisensory integration not only benefits perception by improving precision (reducing variability), but also by reducing bias; a win-win approach.

### Open Questions

While the present work provides valuable insight into the computational mechanism underlying spatial biases in the visual and auditory systems, future research must address several pertinent questions. For example, future studies will need to address the extent of these biases in more complex environments, including more naturalistic settings. For instance, it has been reported that the localization of objects is often biased towards other elements in the visual field [7]. Known as the “landmark attraction effect” [68,69], this effect is particularly relevant for the current paradigm; while this investigation illuminates the biases present in each modality in a sparse visual scene (i.e. with only a fixation cross present at the start of each trial), the question remains as to how biases change as objects that are potential sources of the relevant auditory and visual signals are introduced to the environment.

Finally, we also acknowledge that factors such as stimulus duration, response mechanism, and eye position could potentially influence the magnitude of the biases that emerge in a given modality. For example, one previous study presenting auditory stimuli for a much longer duration than our study (10s) reported greater peripheral biases for eccentric auditory stimuli than what we found in this investigation [14]. Thus, future experiments should seek to systematically manipulate each of these factors to reveal the impact of each variable.

### Conclusion

Nearly every function critical for human survival depends directly on accurate localization of objects and events in the environment. While numerous studies have investigated spatial perception in humans, little consensus exists on whether representation of space in the human brain is accurate or biased, and if the latter is true, how so and why.

The findings of this large-scale study revealed that on average, observers’ localizations are *inaccurate*, as visual localizations show a bias towards the center of space, while auditory localizations show a bias towards the periphery. Even more surprisingly, the observed biases in localization appear to be at least partly due to biases in sensory representations of space as opposed to *a priori* expectations of the spatial layout of objects in the environment (which may be due to a number of non-sensory factors such as the experimental setup, instructions, learning the distribution of stimuli during experiment, etc.).

In real-world settings, objects frequently produce signals that simultaneously stimulate multiple sensory modalities. Considering the opposing biases in the visual and auditory modalities, when visual and auditory stimuli *co-occur* at the *same* spatial location, which bias emerges during localization? The results show that visual bias dominates, however, the magnitude of the central bias in the visual modality is *reduced* when the visual stimulus is integrated with a co-occurring auditory stimulus, thus revealing an additional advantage of multisensory integration: *the reduction of perceptual biases*.
